# Update to the handling of “Preprints” by the Journal of Anesthesia

**DOI:** 10.1007/s00540-020-02885-6

**Published:** 2020-12-15

**Authors:** Michiaki Yamakage

**Affiliations:** Department of Anesthesiology, Sapporo Medical University School of Medicine, Sapporo, Japan

Editor-in-Chief, Journal of Anesthesia

Until now, “the Journal of Anesthesia” and its sister journal “JA Clinical Reports” followed a policy of immediately rejecting all submissions that had posted its preprint version on the web, considering them duplicate submissions. The basis for this strict stance was to raise the bar for quality submissions, as there were endless reports of falsification in research papers by anesthesiologists in Japan.

Preprints are manuscripts that have been uploaded to public servers without going through formal, rigorous peer-review. Preprints are uploaded to the Internet for free, enabling sharing of information among scientists worldwide more quickly than manuscripts published only after completing the traditional peer-review process. Rapidly advancing fields, particularly physics, mathematics, and computer science, have been using this system widely for over two decades, but it is not well established in the fields of medicine. The rationale is that, aside from the irrefutable question of a duplicate submission, as Dr. Kharasch, Editor-in-Chief of Anesthesiology, states [1], the information in preprints that have not been peer-reviewed may be misinterpreted or misused, potentially leading to life-threatening errors in medical practice.

With the emergence of the COVID-19 pandemic that started in Wuhan, China, journals, first in China and now around the globe, have been publishing articles about COVID-19. As specific therapies, effective treatments, and epidemical information for COVID-19 is quite important, publishers of medical journals worldwide have done what they can to post papers on the novel coronavirus as fast as possible and make them freely available. As also explained in the editorial by Dr. Kharasch, Anesthesiology introduced a system for speeding up the peer -review process to that aim. The Journal of Anesthesia adopted the strategy of promptly posting the latest information, primarily by its editors, under the title, “*Anesthesia in the time of COVID-19*,” which is openly accessible. It also created a system to simultaneously send final decisions made by the Editor-in-Chief to the authors, editors, and reviewers.

Nevertheless, this system cannot match the speed of preprints. Springer, the publisher of the Journal of Anesthesia, goes so far as to recommend researchers post preprints [2]. Their stance is based on comments published in Science in 2016, expressing that researchers should post a preprint to one of the applicable servers while simultaneously submitting the manuscript to a journal to undergo peer-review [3]. If the authors have posted a preprint, Springer instructs them to disclose details of the preprint, including the DOI and licensing terms, when submitting their manuscript for publication. If the manuscript is subsequently published, the authors should update the preprint record with reference to the publication.

Consequently, beginning in 2021, “the Journal of Anesthesia” and its sister journal “JA Clinical Reports” will start accepting manuscripts that have posted their preprints on the web. Authors will also be able to resubmit manuscripts that the Journal of Anesthesia and JA Clinical Reports previously rejected because of their preprint version on the server. Please note that manuscripts must include information about existing preprints and that preprints cannot be cited in the manuscript, as they are presumed to have identical content.

I hope that preprints will continue to be used to advance medicine for humankind and that the Journal of Anesthesia and JA Clinical Reports will continue sharing useful information in the field of anesthesiology.
